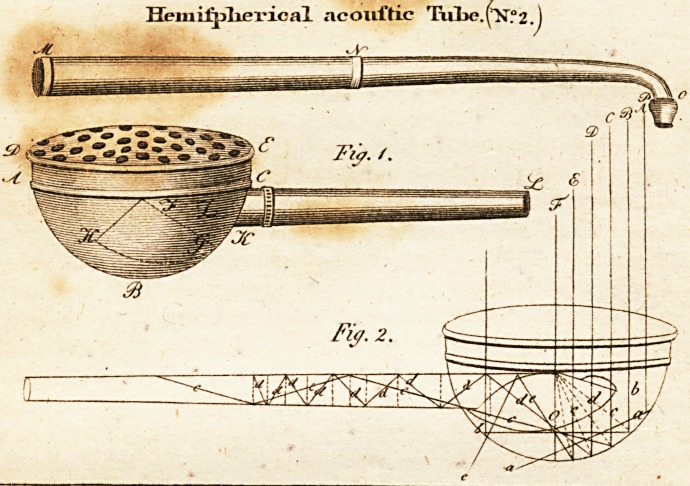# Prof. Arneman, on Acoustic Tubes

**Published:** 1801-07

**Authors:** 


### Prof. ARNEMAN, *on Acoustic Tubes.*

( With an Engraving. )

[ Continued from Vol. IV. p. 379. ]

PROF. ARNEMAN proceeds to examine the different inſtruments that have been propoſed as hearing trumpets. The inſtrument, Tab. II. has been much recommended, though it appears from its conſtruction to be far inferior to that we have already deſcribed. It conſiſts of a hemiſphere made of braſs, ABC, fig. 1. whoſe radius is $= 23'''$ Paris lines, and its diameter conſequently at $AC = 2 + 23''' = 3''$ inches $10'''$ lines. On it is faſtened the cover, which is convex, and perforated with many holes. At CK is an opening $11'''$ in diameter, into which goes a conical conducting tube, $16''$ long, the diameter of which has, at GZ, $= 10'''$, and its opening at OP, $= 4'''$. For greater convenience, the tube may be taken in pieces, as appears at NMC. At OP, or that part of the conducting tube which is applied to the ear, it ends in a top, which is generally made of tin, for the purpoſe of ſhutting the meatus auditorius; but as experience has ſhown, that the continual touching of a metal generally hurts the ear, it would be adviſable to have the top made of ivory, bone, or horn. The conducting tube penetrates into the middle of the hemiſphere, where it is cut off in an oblique direction FG, to which is ſoldered a conical receptacle, the bottom of which is oval, as is ſeen in Fig. 1. HJK. This receptacle is intended for intercepting the ſound, when it is reflected from the interior ſurface of the hemiſphere; but as it ſeems to be placed in a wrong poſition, it is more calculated to confound the ſound, than to conduct it properly. In order to convince the reader of what we have here ſtated, and to ſhow, that from the conſtruction of that inſtrument it is impoſſible it ſhould anſwer its purpoſe, Fig. 2. is added, to demonſtrate how the reflection of the ſound proceeds: It repreſents the ſame acouſtic tube, likewiſe how the rays of ſound fall in, and how they are again reflected; theſe are figured in ABCDEF. It ought here to be firſt conſidered, that the point of concentration tration of the rays of light, as well as of found, fall into the middle of the radius of the inner fuperficies of a globe, which is in this figure at O. This, however, is only the cafe with rays that fall in near the axis; the reft are not fo far propagated, declining the more and more from that point, by which means a confufion and want of diftinction is produced. Hence it appears, that the conical receptacle HIK, intended for the interception of the found, has not the proper form, but that the rays, before they reach it, diverge, and that the ear, for being able to underftand every thing clearly and accurately, ought to be placed at O. The hemifpherical form is likewife of no utility; and by examining the reflection of fome rays, it will be fhown, that it is not proper to convey the found clear and accurate. The ray *f.* for inftance, is reflected in the fame direction as it comes in. A, a ray more diftant from the centre, is reflected at *a*, and confequently does not reach that point where it could be conducted by the intercepting receptacle: The fame may be faid of Bb, which being rebounded againft the oppofite fide, paffes out in the fame direction it fell in. From this it will be underftood, that all rays from A to B are of no ufe at all, but rather confound the found; and thofe that fall in from B to C fpring back in the fame direction they came in, without affecting the ear at all. The ray C is therefore the firft that touches the receptacle, and gets into the conducting tube, though it is likely to be difperfed by being reflected with very acute angles, as is feen in Fig. 2. at ccc. The ray D. rebounds alfo againft the tube, but is reflected with larger angles, increafing by degrees; whence it appears, that the conducting tube muft always be of a determined length, if the found is to be perceived by the ear. The line ddd, fhows the reflection of this ray: The direction in which the ray E is reflected, is feen in ee. This may fuffice to fhow, that only a fmall part of the rays of found, which fall in between C and D, are properly conducted and perceived by the ear, the reft becoming quite ufelefs. This inftrument feems, therefore, not to be properly calculated to convey the found clearly and diftinctly to the organ of hearing; a ftatement which experience has likewife confirmed, as it was ufed by feveral people without the expected fuccefs.

[ To be continued. ]

CRITICAL

## Figures and Tables

**Fig. 1. Fig. 2. f1:**